# Molecular deregulation induced by silencing of the high mobility group protein A2 gene in retinoblastoma cells

**Published:** 2012-10-03

**Authors:** Nalini Venkatesan, Subramanian Krishnakumar, Perinkulam Ravi Deepa, Murali Deepa, Vikas Khetan, M. Ashwin Reddy

**Affiliations:** 1 Department of Ocular pathology, Vision Research Foundation, Sankara Nethralaya, Chennai, India; 2 Department of Biological Sciences, PhD student, Birla Institute of Technology and Science (BITS), Pilani, India; 3 Department of Vitreoretinal and Ocular Oncology, Medical Research Foundation, Sankara Nethralaya, Chennai, India; 4 Department of Ophthalmology, Barts Health, London, England; 5 Department of Pediatric Ophthalmology, Moorfields Eye Hospital, London, England

## Abstract

**Aim:**

To explore the molecular mechanisms deregulated by high mobility group protein A2 (*HMGA2*) gene silencing in retinoblastoma (RB) cells.

**Methods:**

Synthetic anti-*HMGA2* short interfering RNA (siRNA) was used to silence the *HMGA2* gene in cultured Y79 RB cells that were subjected to whole genome microarray analysis. The expression of differentially regulated key genes was confirmed with quantitative reverse-transcriptase polymerase chain reaction (qRT–PCR) in post-silenced RB cell lines (Y79 and WERI Rb1). These deregulated genes were compared for their constitutive expression in primary RB tumors (n=10). Zymographic determination of matrix metalloproteinase (MMP) activity was performed in RB cells. A cell cycle assay and a proliferation assay were performed in post-transfected RB cells.

**Results:**

*HMGA2* gene silencing in cultured RB cells results in reduced cell proliferation and transition in the G1/S phase. The whole genome microarray analysis of *HMGA2* silenced Y79 cells revealed overall upregulation of 1,132 genes (≥1.0 fold) and downregulation of 1,562 genes (≤ −1.0 fold). Specific quantitative pathway analysis of the deregulated genes (using Biointerpreter) revealed 150 upregulated genes and 77 downregulated genes (≥1.0 fold) involved in vital pathways, namely, mitogen-activated protein kinase, Janus kinase/signal transducers and activators of transcription, Ras pathway, Ras-induced extracellular signal-regulated protein kinases 1 and 2, and tumor protein p53. The differential expression of genes obtained from microarray analysis (Homo sapiens ELK1, member of ETS oncogene family [*ELK1*], Homo sapiens cyclin-dependent kinase 6 [*CDK6*], Homo sapiens E2F transcription factor 4, p107/p130-binding [*E2F4*], Homo sapiens G-2 and S-phase expressed 1 [*GTSE1*], Damage-regulated autophagy modulator [*DRAM*], Homo sapiens cadherin 1, type 1,E-cadherin (epithelial) [*CDH1*], Homo sapiens snail homolog 1 (Drosophila) [*SNAI1*], Homo sapiens matrix metallopeptidase 2 [*MMP2*], and Homo sapiens matrix metallopeptidase 9 [*MMP9*]) was confirmed with quantitative reverse-transcriptase polymerase chain reaction in post-silenced RB cells. Zymographic analysis revealed that the increase in *MMP* mRNA expression in the post-silenced RB cells did not correlate with corresponding enzyme activity.

**Conclusions:**

Our study revealed molecular regulatory changes induced by *HMGA2* silencing in RB cancer cells, offering mechanistic insights into the anticancer potential. *HMGA2* may be considered a promising candidate for gene silencing therapy in RB.

## Introduction

Retinoblastoma (RB), a common primary intraocular tumor, occurs in infants and children with a relative incidence of 3% of all pediatric tumors worldwide [1]. Newer molecules and pathways have to be identified for designing novel targeted therapies in managing RB to avoid enucleation and to prevent metastasis [2]. EpCAM, Stathmin, and Connexin 46 are recent examples of newer therapeutic and drug delivery targets in RB [3–7]. The expression of another promising molecular candidate, high mobility group protein A2 (HMGA2), was reported in RB by our group [8]. The strong correlation between HMGA2 expression and tumor invasiveness has prompted further research on this molecule and related pathways [8,9].

The HMGA family consists of HMGA1a, HMGA1b, HMGA1c, and HMGA2 [10,11]. HMGAs are small non-histone chromosomal proteins, characterized by highly conserved DNA-binding motifs called “AT-hooks” and an acidic tail [12]. HMGA1 (HMGA-1/Y) and HMGA2 (HMG1-C) have similar functions and are found relatively abundantly in the early embryo, where cells are proliferating rapidly [13,14].

However, the *HMGA2* gene is not detectable in adult human tissues where it is probably completely silenced [15,16]. The ectopic expression of *HMGA2* in RB [9] and the clinicopathological correlations of HMGA2 in primary RB tissues [8] suggest its role in the genesis and maintenance of the transformed phenotypes [17]. Reexpression of the *HMGA2* gene was observed in the cells of many human malignancies such as breast and non-small lung cancers [18], pancreatic carcinoma [19], breast cancer [20], squamous cell carcinomas [21], and myeloproliferative disorders [22]. HMGA2 is being studied for its oncogenic properties [23,24], stem cell self-renewal [25,26], DNA damage response [27], and tumor cell growth and differentiation [28–30]. However, the precise role of HMGA2 in malignant transformation and the gene’s regulation of tumorigenesis are still not clear. Previous studies on *HMGA2* gene silencing inhibited Ras-induced transformation of thyroid cells resulting in growth inhibition and increased apoptosis of liposarcoma cells [31,32]. Using a nude mice model of retinoblastoma, Ono et al. suggested a potential role for HMGA2 derepression in the tumorigenesis of retinoblastoma [9].

In this study, we have established the suppression of cell proliferation in cultured RB cells of differing tumor aggressiveness (Y79 and WERI Rb1) using the *HMGA2* gene-silencing technique. The DNA binding sites in the *HMGA2* gene are being identified [33,34] with possible implications for developing DNA-based therapeutics (aptamers). However, molecular understanding of HMGA2-mediated cell signaling is limited. Here, we present the key findings on the molecular effects of *HMGA2* gene silencing in cell signaling, apoptotic, and cell adhesion regulation in RB. The deregulated genes in the post-silenced RB cells were compared with primary tumors for constitutive expression levels.

## Methods

The study was reviewed and approved by the local ethics committee of our institute, and the committee deemed that the study conformed to the generally accepted principles of research, in accordance with the Helsinki Declaration.

### Cell culture

Human RB cell lines (Y79, WERI Rb 1, RIKEN BioResource center, Ibaraki, Japan) were used in the study. Cells were cultured in Roswell Park Memorial Institute 1640 medium (RPMI; Gibco-BRL, Rockville, MD) supplemented with 10% heat-inactivated fetal calf serum (FBS; Gibco-BRL), 0.1% ciprofloxacin, 2 mM L-glutamine, 1 mM sodium pyruvate, and 4.5% dextrose (Sigma Aldrich, St. Louis, MO) and grown in suspension at 37 °C in 5% CO_2_.

### Tumor samples

The present study includes tumor samples collected from ten enucleated eyeballs of patients with RB (2010–2011). The RB sections were reviewed and graded microscopically by an ocular pathologist. The tumor samples were recorded for their clinicopathological features based on the predominant pattern of differentiation and tumor invasion of the choroid, optic nerve, or orbit (with or without metastasis) following the International Intraocular RB Classification (IIRC; Table 1) [35].

**Table 1:** Clinicopathological features of the primary RB tumors following the International Intraocular Retinoblastoma Classification (IIRC) with gene expression (by qRT–PCR).

**S/N**	**Age/Sex**	**Clinico pathological features**	**Log 2 ratio fold change**						
			**ELK1**	**GTSE1**	**CDK6**	**E2F4**	**DRAM**	**CDH1**	**SNAI1**
1	4/M	OS:PD tumor seen in iris surface, trabecular meshwork, diffuse CI with measuring >3 mm thickness, prelaminar, laminar invasion, invasion of anterior & middle portion of sclera .	++	-	++	++	-	NS	+
2	3/F	OS: WD with formation of fleurettes, prelaminar invasion of ON.	++	++	++	++	+	NS	++
3	2/M	OS:WD massive CI >3 mm, tumor cells invading the anterior, middle and posterior border of sclera with spill over into the orbital tissue.	++	++	++	++	+	+	NS
4	4/M	OD:PD, there is full thickness diffuse CI measuring >3 mm, tumor cells touching the anterior border of scelra, prelaminar invasion of ON	++	++	++	++	+	NS	++
5	8/ F	OD:UD, tumor cells adherent to iris surface, invasion of ciliary process, diffuse full thickness CI >3 mm, invasion of prelaminar, laminar & post laminar portion of ON,outer margin of sclera.	++	++	++	++	+	NS	++
6	6mon/F	OS;MD, CI measuring >3mm, prelaminar invasion and laminar invasion of the ON.	++	++	++	++	+	+	++
7	3/M	OS:PD, focal CI measuring <3 mm.	++	++	++	++	+	NS	++
8	5/M	OD:UD prelaminar invasion of ON	++	-	++	++	+	NS	++
9	2/F	OD: PD, tumor cells in iris surface, full thickness CI measuring 10 mmx7 mm.Sclera invasion.	++	++	++	++	+	NS	++
10	1/F	OS: WD, focal choroid invasion measuring <3mm.	++	++	++	++	+	NS	++

F:female; M:male; MD:moderately differentiated; PD:poorly differentiated; WD:well differentiated; OD:right eye; OS:left eye; CI:choroidal invasion; ON:optic nerve; **++**: Up-Regulated genes; **+**: Down-Regulated; **-**: Negative; NS: Not significant fold change

### Short interfering RNA sequences

The transfection protocol was performed using three different short interfering RNA (siRNA) sequences targeting the *HMGA2* gene. Human *HMGA2* siRNA.1 (Hs_HMGA2_6 catalog number SI03029929: Forward strand: 5′-CGG CCA AGA GGC AGA CCU ATT-3′ and the reverse strand: 5′-UAG GUC UGC CUC UUG GCC GTT-3′), *HMGA2* siRNA.2 (Hs_HMGA2_7 catalog number SI03067393: Forward strand: 5′-GCG GCG GCA GCC UAA GCA ATT-3′ and the reverse strand: 5′-UUG CUU AGG CUG CCG CCG CTG-3′), and scrambled siRNA (catalog number 1,022,563) *HMGA2* siRNA.3 (1146–1164; catalog number 1,027,423, forward strand: 5′-CGC CAA CGU UCG AUU UCU-3′ and the reverse strand: 5′-GCG GUU GCA AGC UAA AGA-3′), were used in this study. The *HMGA2*-specific siRNAs and the scrambled siRNA were purchased from Qiagen (Santa Clara, CA).

### Transfection

About 1×50,000 cells were plated per well (12 well plates), and allowed to grow for 24–36 h (until they were 40%– 60% confluent) and incubated with 0.5 ml of antibiotic-free media, 0.5 ml of complete media containing 200 nM *HMGA2*-specific siRNAs (Qiagen) plus 5 µl of Lipofectamine transfection reagent (Invitrogen, Paisley, Germany) for 24 h, 48 h, and 72 h. The cells were harvested and processed for quantitative reverse-transcriptase polymerase chain reaction (qRT–PCR) and western blot analysis. The same protocol was applied using 200 nM non-target scrambled siRNA (Qiagen) for the cell proliferation assay.

### Relative *HMGA2* gene expression in pre- and post-silenced cells with qRT–PCR

To quantify the *HMGA2* RNA expression in untreated and siRNA-treated RB (Y79, WERI Rb1) cells, tumor tissues, the total RNA was extracted with the guanidine isothiocyanate and chloroform method (TRI Reagent; Sigma Aldrich). Cells were harvested from cultures and collected in RNase-free vials. To the pellet, 1 ml of TRIzol reagent (TRI Reagent) was added, vortexed vigorously for 2 min, and incubated at room temperature for 5 min. Later, 0.5 ml of chloroform was added to the solution and mixed well for 15 s and centrifuged. The aqueous layer that contains RNA was transferred to new vials. Then 0.5 ml of isopropanol was added and incubated at room temperature for 10 min. After centrifugation, the supernatant was discarded, and 0.5 ml of 75% ethanol was added, mixed well, and centrifuged. Then the supernatant was discarded. The pellet was air dried at room temperature for 2 min and reconstituted in 25 µl of RNase-free water. All centrifugations in the RNA extraction were performed at 15,000 × *g* for 10 min at 4 °C. All RNA samples were treated with TURBO DNase (Ambion, Genetix Biotech Asia Pvt. Ltd., Chennai, India). For all samples, 1 μg of total RNA was used to synthesize first-strand cDNA with reverse transcriptase (Sensiscript II; Qiagen, Santa Clara, CA) and random primers. The cDNA synthesis was performed at 37 °C for 60 min followed by heat inactivation at 95 °C for 10 min. Gene expression assays for *HMGA2* (Hs00171569_m1) and endogenous control, glyceraldehyde-3-phosphate dehydrogenase (*GAPDH*; Hs99999905_ml), were obtained from Applied Biosystems (Lab India, Chennai, India).

*HMGA2* gene expression was normalized with *GAPDH* expression, which was measured using predeveloped assay reagents (Applied Biosystems). The final volume for each PCR was 20 μl including 1 μl (100 ng) of the investigated sample 1× Universal PCR Master Mix (TaqMan, ABI Applied Biosystem) for *HMGA2* gene expression according to the manufacturer’s instructions. Gene expression in each sample was analyzed in triplicate. The PCR for *HMGA2* gene expression using TaqMan probes was performed as follows: 2 min at 50 °C, 10 min at 95 °C, and 40 cycles of 15 s at 95 °C, plus 1 min at 60 °C. PCR for the other genes was performed as follows: commercial software (SDS ver. 1.3; ABI) was used to calculate ΔΔC_t_ [36] relative expression values for these genes, which were normalized to the *GAPDH* endogenous control.

### Western blot analysis

The post-silenced Y79 and WERI Rb1 cells were tested for HMGA2 protein extraction. Cells were treated with 5% perchloric acid. The proteins were precipitated with equal volumes of cold acetone at 20 °C overnight. The precipitate was collected and centrifuged at 20,000 × *g* for 15 min at 4 °C and washed with acetone at 4 °C. The dried proteins were dissolved directly in sample buffer (2% sodium dodecyl sulfate, 0.0625 M Tris-HCl [pH 6.8], 5 mM EDTA, and 10% glycerol). The protein was resolved by using 18% acrylamide gel. The separated proteins were electrophoretically transferred to the nitrocellulose membrane at 100 V for 1 h. The blots were incubated with human HMGA2 primary antibody (1:1,000; catalog no. SC-30223) and human histone H1 (1:1,000; catalog no. SC-8030; Santa Cruz Biotechnology, Santa Cruz, CA) overnight at 4 °C followed by anti-rabbit horseradish peroxidase-conjugated secondary antibody (1:5,000; catalog no. SC-2004; Santa Cruz Biotechnology) incubation for 2 h. To determine the p53, p21, and β-actin proteins, the total protein cell lysate of the post-silenced Y79 and WERI Rb1 cells was extracted using lysis buffer containing 50 mM Tris-HCl (pH 7.6), 5 mM EDTA, 150 mM sodium chloride, 0.1% phenylmethanesulfonyl fluoride (PMSF), and 250 ml of 1 mg/ml protease inhibitor cocktail on ice. A total protein of 25 μg was resolved on by using 12% sodium dodecyl sulfate–PAGE. The separated proteins were electrophoretically transferred to the nitrocellulose membrane at 100 V for 1 h. The blots were incubated with human p53 primary antibody (1:1,000; catalog no. SC-126), human p21 (1:1,000; catalog no. SC-6246; Santa Cruz Biotechnology), and β-actin (1:5,000; clone no. AC 74; Sigma Aldrich) overnight at 4 °C followed by anti-mouse horseradish peroxidase-conjugated secondary antibody (1:5,000 for p53 and p21 determination and 1:10,000 for β-actin determination; catalog no. SC-2500; Santa Cruz Biotechnology) incubation for 2 h. After intermittent washes with Tween Tris-buffered saline, the membranes were subjected to the chemiluminescence detection method (SuperSignal West Femto Maximum Sensitivity Substrate; Fisher Scientific, Pittsburgh, PA). To derive the HMGA2 concentration in the individual samples, the intensity of the bands was measured using Quantity One, version 4.7 software in GS-800 calibrated Densitometer (Bio-Rad, Gurgaon, India) and normalized with the respective histone expression. A similar protocol was followed to determine the concentration of p53 and p21, and normalized with the respective β-actin expression.

### Flow cytometric analyses

The post-silenced Y79 and WERI Rb1 cells were tested for caspase 3 expression. Cells were harvested, washed, and resuspended in ice-cold phosphate buffered saline (PBS; 8 g Nacl, 0.2 g KCl, 1.44 g, Na_2_HPO_4_, 0.24 g KH_2_PO_4_, pH 7.4). Mouse monoclonal primary antibody against caspase 3 (1:50 diluted in fetal bovine serum, catalog no. SC-7272; Santa Cruz Biotechnology) was used and incubated for 2 h at 4 °C. Following incubation, cells were washed three times with ice-cold PBS. Fluorescein isothiocyanate–conjugated anti-mouse secondary antibody (1:750, catalog no. SC-2500; Santa Cruz Biotechnology) was used and incubated for 1 h at 4 °C in the dark. Later, the cells were washed three times with ice-cold PBS. Cells were analyzed using a FACSCalibur flow cytometer (BD Biosciences, San Jose, CA), with the CellQuest software program (BD Biosciences).

### Cell cycle analysis

RB cells (Y79, WERI Rb1) were harvested after transfection with siRNA for 48 h. Cells were fixed for 30 min with 70% cold ethanol, washed twice with cold PBS, and then incubated in PBS buffer 100 μg/ml RNase A for 30 min. Propidium iodide 5 μg/ml was added, incubated for 10 min, and cells detected with a flow cytometer (FACSCalibur, Becton-Dickinson, Franklin Lakes, NJ).

### Proliferation assay

Transfected Y79 and WERI Rb1 cells (5,000 cells per well) were plated in 96 well plates on day 0. On day 1, cells were incubated with 200 nM *HMGA2* specific siRNA.1 (Hs_HMGA2_6; Qiagen) plus 0.5 µl of Lipofectamine transfection reagent (Invitrogen) for 24 h. This was repeated on days 2 and 3. On the days from 1 to 3, serum-free RPMI medium containing 10 µl of 5 mg/ml 3-(4,5- Dimethylthiazolyl)2,5diphenyltetrazolium bromide (MTT) was added to the wells, and the cells were incubated at 37 °C for 4 h. Then 100 µl of MTT solubilization solution dimethyl sulfoxide (DMSO, Sigma Aldrich, St Louis, MO) was added, and the cells were incubated at 37 °C for 10 min. Colorimetric measurements were made using a spectrophotometer (Beckman Coulter India Private Ltd, New Delhi, India) at 562 nm, and the background was subtracted at 650 nm. The assay was performed in triplicate with and without scrambled siRNA as controls.

### Whole genome complementary RNA microarray analysis in *HMGA2*–silenced Y79 cells

Total RNA used for the microarray analysis was isolated from siRNA-treated and -untreated Y79 cells using TRIzol Reagent (Invitrogen, Carlsbad, CA) and treated with TURBO DNase (Ambion, Genetix Biotech Asia Pvt. Ltd.) to remove the DNA. The RNA samples (10 μg each) in a 50-μl reaction were treated with 1 μl of TURBO DNase (2 U) in 1× TURBO DNase buffer at 37 °C for 30 min. After the incubation, the RNA sample was extracted with phenol/chloroform to inactivate TURBO DNase. The Low RNA Input Linear Amplification Kit PLUS (Agilent Technologies Genotypic, Bangalore, India) was used to generate fluorescent complementary RNA (cRNA). T7 RNA polymerase was used in this method, which simultaneously amplifies target material and incorporates Cy3-labeled cytidine tri-phosphate. Qiagen’s RNeasy mini spin columns were used to purify the amplified cRNA samples, and the samples were then hybridized to the Human Whole Genome 44 K oligo Microarray for 17 h at 65 °C, as recommended by the manufacturer (Agilent Technologies). Data analysis was done using GeneSpring GX version 10 (Agilent Technologies). Agilent Feature Extraction software (G25677AA; Agilent Technologies) was used to analyze the microarray data.

### Gene expression analysis in *HMGA2*-silenced retinoblastoma cells and primary retinoblastoma tissues with qRT–PCR

Following the microarray analysis, a panel of genes involved in cell signaling, apoptosis, and cell adhesion mechanisms was selected for further confirmation with qRT–PCR. The differential expression of these genes was investigated in post-silenced Y79 and WERI Rb1 cells, and compared with control primary RB tissues.

The extraction of total RNA and the cDNA conversion was performed as described above. The final volume for each PCR was 20 μl, including 1 μl (100 ng) of the investigated sample 1× Universal RT^2^ Real Time TM SyBr Green/ROX PCR Master Mix (catalog no. 330,520; ABiosciences, New Delhi, India) used according to the manufacturer’s instructions. The primer sequences used in the gene expression study are provided in Table 2.

**Table 2:** The primer sequences used for validation of the key genes in the primary retinoblastoma tumors by qRT–PCR.

**Gene name**	**Sense/Antisense: Sequences**
ETS oncogene family (ELK1)	FP: 5′GAAGAATCACACCCTTGGAA3′
	RP: 5′GACAAAGGAATGGCTTCTCA 3′
G-2 and S-Phase expressed1(GTSE1)	FP: 5′ACGTGAACATGGATGACCCTA3′
	RP: 3′GTTCGGGAACCGGATTATTTA5′
cyclin dependent kinase 6 (CDK6)	FP: 5′CTGAATGCTCTTGCTCCTTT3′
	RP: 5′AAAGTTTTGGTGGTCCTTGA3′
E2Ftranscriptionfactor4,p107/p130binding(E2F4)	FP: 5′GGGCAGAAGAAGTACCAGATTCA3′
	RP: 5′GCTCCATGCCTCCTTGTTCA3′
v – crk sarcoma virus CT10 oncogene homolog(avian; CRK) transcript variant	FP: 5′CCGGGACAAGCCTGAAGAGC3′
	RP: 5′GGCCCACCCAGTGGCTGTGG3′
CDH1	FP: 5′TCGACACCCGATTCAAAGTGG3′
	RP: 5′TTCCAGAAACGGAGGCCTGAT3′
SNAIL homolog 1 (Drosophila; SNAI1)	FP: 5′TATGCTGCCTTCCCAGGCTTG3′
	RP: 5′ATGTGCATCTTGAGGGCACCC3′
MMP 2	FP: 5′AGATCTTCTTCTTCAAGGACCGGTT3′
	RP: 5′GGCTGGTCAGTGGCTTGGGGTA3′
MMP 9	FP:5′GCGGAGATTGGGAACCAGCTGTA3′
	RP: 5′GACGCGCCTGTGTACACCCACA3′

### Activity staining of matrix metalloproteinase with zymography

The transfected cells (Y79 and WERI Rb1) were collected and washed thrice with phosphate buffer. When the protein lysate was prepared, 200 μl of 10 mg/ml phenylmethanesulfonyl fluoride (P7626; Sigma Aldrich) and 10 μl of 1 mg/ml protease inhibitor cocktail (Sigma Aldrich) were added, and the samples were sonicated (VirSonic; Virtis, SP Industries Inc., Gardiner, NY) three times for 10 s each, on ice. The samples were then incubated in 4 °C for 15 min and centrifuged under cooling conditions (REMI C-24 Remi High Speed Cooling Centrifuge, Thane, India) at 2,655 × *g* for 5 min. The supernatant was collected, and the proteins were estimated with the Lowry method. Ten-percent sodium dodecyl sulfate–PAGE gels incorporated with 1 mg gelatin (Merck Biochemicals, MSD, Pharmaceuticals Private Ltd, Gurgaon, India) was prepared. About 50 μg of each sample with equal volume of Native Loading Buffer (0.1 mg bromophenol blue, 2 ml glycerol, 2.5 ml 0.5 M Tris, pH 7.4) were loaded and run at 150 V for 90 min. The gels were renatured in Tris-HCl (pH 6.8) and washed thrice in 5.5 ml milli Q water for 15 min, left overnight in low salt collagenase buffer (LSCB) buffer (50 mM Tris, 0.2 M NaCl, 5 mM CaCl_2_) for three changes of 30 min each in 2.5% Triton X-100, and then washed with milli Q water, 0.02% Brij 35, and 0.02% sodium azide (pH 7.6) at 37 °C. The gels were then stained with 0.5% Coomassie Blue (Sigma Aldrich) for 90 min and destained in 10% acetic acid to reveal zones of digestion.

### Statistical analysis

For microarray analysis, the statistical *t* test and p value were determined based on a volcano plot using the Benjamini and Hochberg algorithm. A p value ≤0.05 was considered significant for change in gene expression. Log_2_ transformed values of gene expression changes showing ≥1.0 fold were considered upregulation, while a ≤1.0 fold change was considered downregulation in gene expression. A minimum of three replicates was performed for all cell culture experiments to derive the standard deviation (SD).

## Results

### Optimization of short interfering RNA–mediated downregulation of *HMGA2* in retinoblastoma (Y79) cell lines

**Figure 1:**
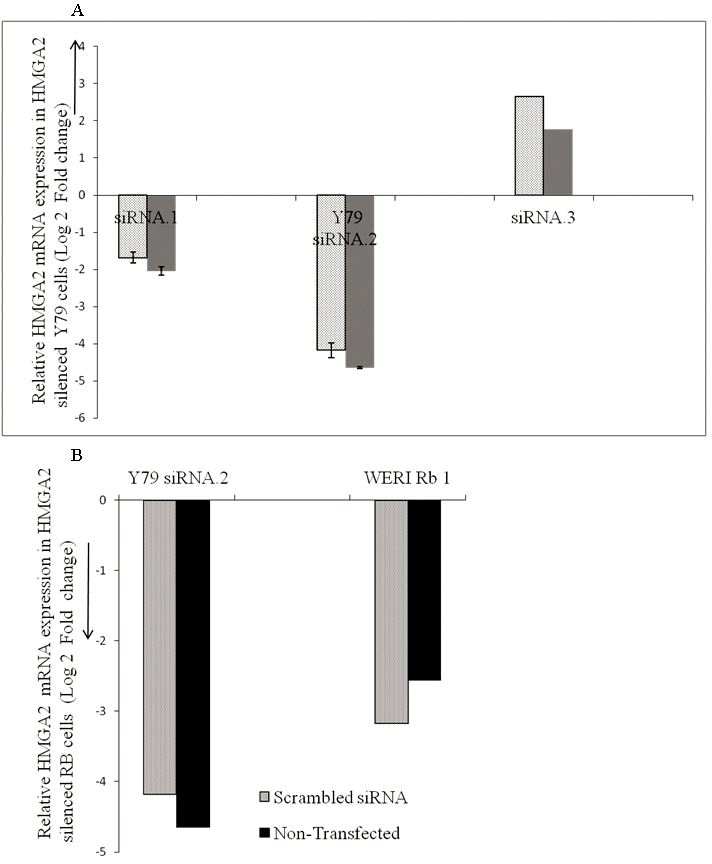
Effect of small interfering RNA on the expression of *HMGA2* in RB Y79 cells in vitro. **A**: The mRNA levels of high mobility group A2 (*HMGA2*) in RB cells (Y79) treated with *HMGA2* short interfering (si) RNA, namely, the siRNA.1 (Hs_HMGA2_6) sequence, led to a 4.65 log_2_ ratio decrease, siRNA.2 (Hs_HMGA2_7) led to a decrease of 2.0, the siRNA.3 sequence led to an increase of 1.76 log_2_ ratio when compared with *HMGA2* mRNA levels in control (solid bar) and to a decrease of a 1.68 log_2_ ratio, 4.175 log_2_ ratio, an increase of a 2.65 log_2_ ratio, respectively, to cells treated with scrambled (SCR) siRNA (dotted bars) at the end of 48 h. The error bars represent the standard deviation of triplicate values. **B**: The mRNA levels of high mobility group A2 (*HMGA2*) in Y79 cells treated with HMGA2 short interfering (si) RNA [siRNA.1 (Hs_HMGA2_6) sequence] led to a decrease of a 4.65 log_2_ ratio, in WERI Rb1 led to a decrease of a 2.56 log_2_ ratio when compared with *HMGA2* mRNA levels in RB control cells (solid bar) and to a decrease of a 4.175 log_2_ ratio, a 3.17 log_2_ ratio, respectively, in RB (Y79, WERI Rb1) cells treated with scrambled (SCR) siRNA (dotted bars at the end of 48 h). The error bars represent the standard deviation of triplicate values.

**Figure 2:**
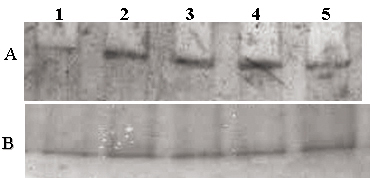
Effect of small interfering RNA on the expression of HMGA2 in retinoblastoma (Y79, WERI Rb1) cells in vitro. **A**: western blot analysis demonstrates markedly reduced HMGA2 expression in Y79 cells treated with *HMGA2*-siRNA (lane 1), strong expression of HMGA2 in non-transfected Y79 cells (lane 2), strong expression in Y79 cells treated with scrambled siRNA (lane 3), strong expression of HMGA2 in non-transfected WERI Rb1 cells (lane 4), and markedly reduced HMGA2 expression in WERI Rb1 cells treated with *HMGA2*-siRNA (lane 5). **B**: western blot analysis demonstrates expression of histone (normalization control) in Y79 cells treated with *HMGA2*-siRNA (lane 1), in non-transfected Y79 cells (lane 2), Y79 cells treated with scrambled siRNA (lane 3), in non-transfected WERI Rb1 cells (lane 4), and in WERI Rb1 cells treated with *HMGA2*-siRNA (lane 5).

Initially, the *HMGA2* gene silencing protocol was optimized in cultured RB cells. Using qRT–PCR analyses, we found that over a period of 48 h, transfection with the siRNA.1 (Hs_HMGA2_6) sequence led to a −4.65 log_2_ ratio decrease, while the other sequences, siRNA.2 (Hs_HMGA2_7) and siRNA.3 sequence, led to a log_2_ ratio of −2.0 and 1.76 decrease, respectively, only when compared with and without scrambled siRNA as controls in RB cells (Y79; Figure 1A). This result when using the siRNA.1 (Hs_HMGA2_6) sequence in the study is consistent with the western blot analysis of the same in RB cells (Y79; Figure 2).

### Comparison of *HMGA2* gene mRNA and protein expression in pre- and post-silenced Y79 and WERI Rb1 cells

The effect of *HMGA2* gene silencing in the pre- and post-silenced Y79 and WERI Rb1 cells using the siRNA.1 (Hs_HMGA2_6) sequence showed decreased expression of the log_2_ ratio of −4.65 and −3.17, respectively. The protein expression of HMGA2 was confirmed with western blot analysis (Figure 1B).

### Arrest of cell cycle progression in retinoblastoma cells (Y79, WERI Rb1) with *HMGA2* gene silencing

**Figure 3:**
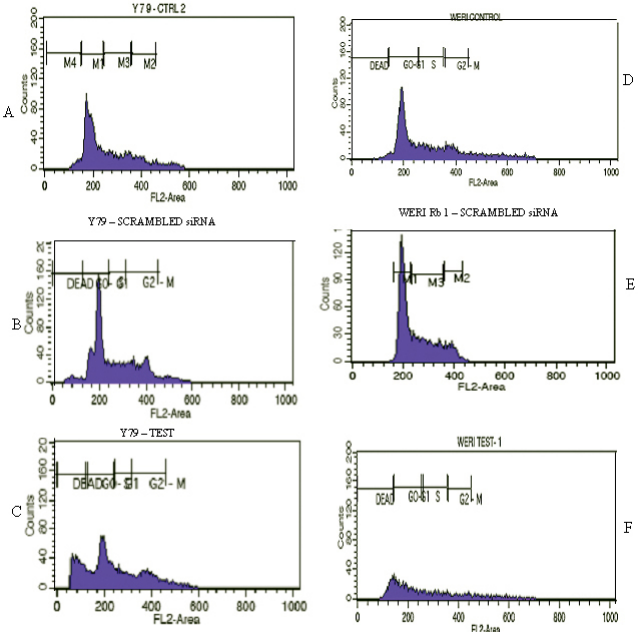
*HMGA2* short interfering RNA treatment results in the marked arrest of the cell cycle phase. **A**: Untreated Y79 cells. **B**: Scrambled siRNA treated Y79 cells. **C**: *HMGA2* siRNA treated Y79 cells showing marked **G**:_0_/**G**:_1_ and **S**: phase cell cycle arrest compared to the control cells. **D**: Untreated WERI Rb1 cells. **E**: Scrambled siRNA treated WERI Rb1 cells. **F**: *HMGA2* siRNA-treated WERI Rb1 cells show marked **G**:_2_/M phase cell cycle arrest compared to the control cells.

The effect of *HMGA2* gene silencing on modulating RB cell growth was studied using a cell cycle assay after transfection with *HMGA2* siRNA (Table 3). Cell cycle distribution was assessed with flow cytometry. Compared to the untreated cells, the G_0_/G_1_ and S phases in the post-transfected Y79 cells, and the G_0_/G_1_, S, and G_2_/M phases in post-transfected WERI Rb1 cells showed marked cell cycle arrest (Figure 3).

**Table 3:** Cell cycle analysis in treated and control Y79 and WERI Rb1 cells.

**Cell cycle/group**	**G0/G1 (%)**	**G2/M (%)**	**S (%)**
Y79 (Control)	56.15±0.33	13.00±0.97	25.73±0.48
Y79 (Scrambled siRNA)	50.82±0.71	26.23±0.22	14.70±0.61
Y79 (HMGA2 siRNA Treated)	39.53±0.57	19.57±0.5	12.64±0.37
WERI Rb1 (Control)	52.05±0.41	13.58±0.57	19.20±0.18
WERI Rb1 (Scrambled siRNA)	54.42±0.67	9.86±0.51	34.28±0.0
WERI Rb1 (HMGA2 siRNA Treated)	42.19±0.91	9.84±0.25	15.65±0.69

### Suppression of retinoblastoma cell proliferation by *HMGA2* gene silencing

**Figure 4:**
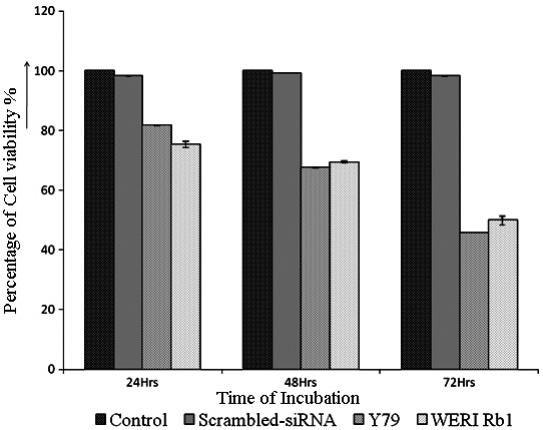
*HMGA2* short interfering RNA treatment decreases the proliferation of RB (Y79, WERI Rb1) cells. The Y79 cells were treated with high mobility group A2 (*HMGA2*) short interfering (si)RNA, and the cell proliferation was assessed at 24, 48, and 72 h using 3-(4,5-dimethylthiazol-2-yl)-2,5-diphenyltetrazolium bromide (MTT) assay. The cell viability decreased to 81.7%, 67.5%, and 45.5% in the Y79 cell line, and to 75.4%, 69.4%, and 49.9% in the WERI Rb1 cell line when compared to Y79 cells that were not treated with siRNA. The error bars represent the standard deviation of triplicate values.

The effect of *HMGA2* gene silencing on modulating cell proliferation was studied using an MTT assay. The MTT assay in the RB (Y79, WERI Rb1) cells treated with the anti-*HMGA2* siRNA, scrambled siRNA (a control for non-specific effects of siRNA treatment on cell growth), and the untreated Y79 cells at the end of 24 h, 48 h, and 72 h resulted in a significant decrease in cell proliferation to 81.7%, 67.5%, and 45.5% in Y79 cells and 75.4%, 69.4%, and 49.9% in WERI Rb1 cells, respectively (Figure 4). There was no significant difference in the cell proliferation rate between the scrambled siRNA and untreated Y79 cells.

### CDNA microarray analysis

**Figure 5:**
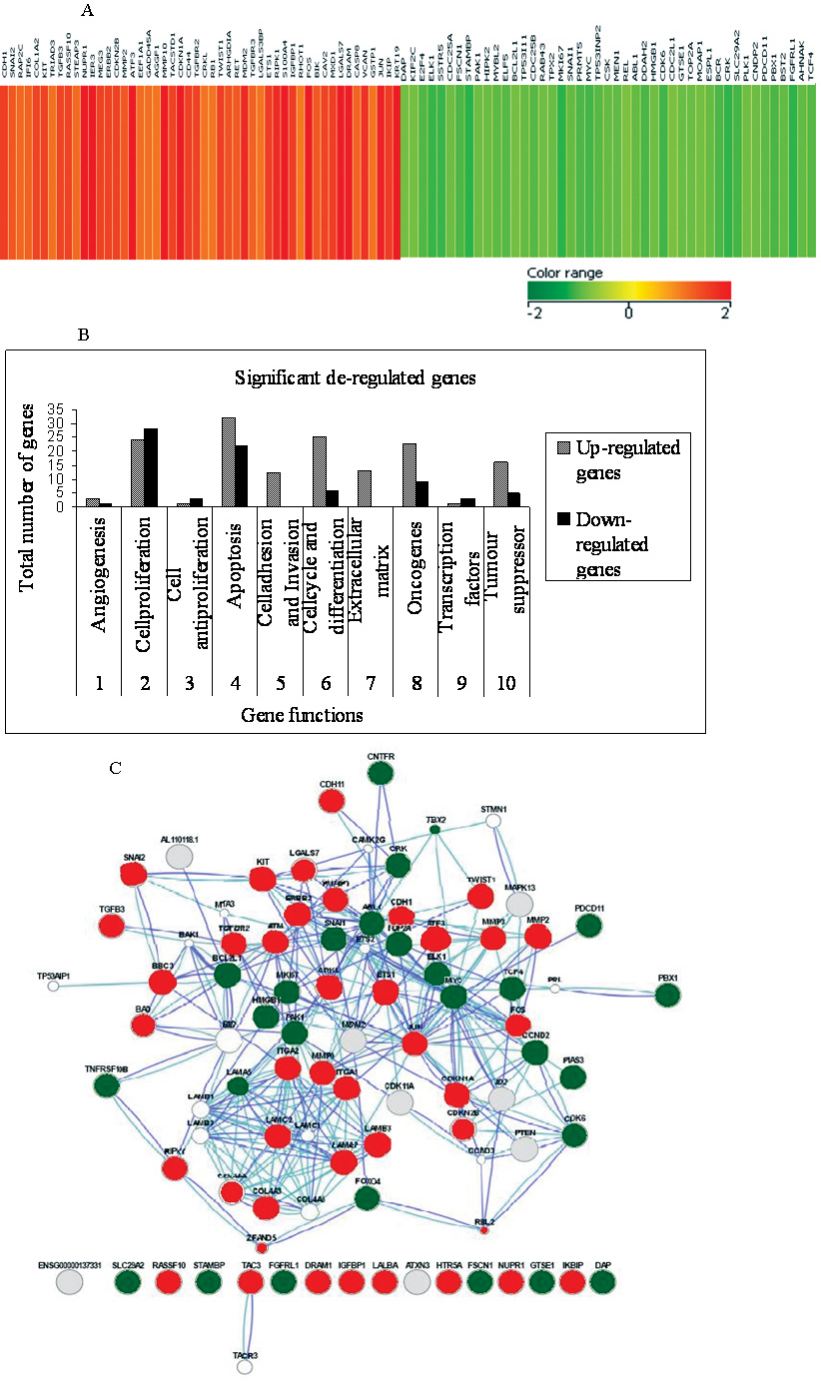
*HMGA2* short interfering RNA treatment leads to changes in the gene expression profile in Y79 cells. **A**: The heat map represents the expression profile of 100 genes differentially modified in response to knockdown of high mobility group A2 (*HMGA2*) in Y79 cells compared to untreated cells. The horizontal lines represent the relative fold change in the expression of individual genes modified by the *HMGA2*-short interfering siRNA. Red and green indicate increased and decreased expression, respectively, relative to non-silenced control Y79 cells. **B**: Functional grouping of upregulated genes and downregulated genes in Y79 cells treated with *HMGA2* short interfering siRNA. The functional grouping of all the distinct gene identification for their known biologic function was done according to gene ontology. **C**: The interlinking of distinct genes identified for their known biologic function are drawn using the GeneMANIA tool.

The genome-wide expression of anti-*HMGA2* siRNA treated and untreated Y79 cells were analyzed to probe the genes regulated by the *HMGA2* gene. As a result of the anti-*HMGA2* siRNA treatment in Y79 cells, a total of vital 227 gene transcripts involved in various cellular functions were modulated, which includes 150 upregulated (66.07%) and 77 downregulated (33.9%). Significantly, dysregulated pathways were identified using several databases such as Biologic Pathway Exchange (BioPAX) pathways from Biocarta, Human Protein Reference Database (HPRD), Reactome, KEGG, and NCI-cGAP, by querying the differentially expressed gene list against all the genes annotated with pathway information in the microarray. A total of 100 upregulated and downregulated genes of interest are shown (Figure 5A-C). Gene descriptions for some of the key genes shown are presented in Table 4 and Table 5. The data discussed in this publication have been deposited in NCBI's Gene Expression Omnibus (GEO) [37] and are accessible through GEO Series accession number GSE31687.

**Table 4:** Up - Regulated Genes in post HMGA2 silenced in Y79 cells (cDNA microarray analysis).

**Gene classification**	**Gene Description (Homo sapiens, gene, mRNA)**	**Gene symbol**	**Fold change**	**Gene Bank Accession**	**Chromosome location**
Angiogenesis	Lectin, galactoside-binding, soluble, 3 binding protein	GALS3BP	1.1	NM_005567	17q25.3
	Angiogenic factor with G patch and FHA domains 1	AGGF1	1.093	NM_018046	5q13.3
Cell cycle, differentiation	Transforming growth factor, beta 3	TGFB3	1.511	NM_003239	14q24.3
	Activating transcription factor 3, transcript variant 2	ATF3	2.448	NM_004024	1q32.3
Cell proliferation and invasion	Matrix metallopeptidase 10 (stromelysin 2)	MMP10	2.958	NM_002425	11q22.2
	p53 binding protein (mouse), transcript variant	MDM2	1.764	NM_002392	12q15
	v-fos FBJ murine osteosarcoma viral oncogene homolog	FOS	2.76	NM_005252	14q24.3
	MMP 2 (72 kDa gelatinase A, 72 kDa type IV collagenase)	MMP2	1.338	NM_004530	16q12.2
Anti – proliferation	Maternally expressed 3	MEG3	1.53	NR_002766	14q32.2
Apoptosis	Apoptosis-related cysteine peptidase, transcript variant C	CASP8	1.22	NM_033356	2q33.1
	BCL2-interacting killer (apoptosis-inducing)	BIK	1.319	NM_001197	22q13.2
	Damage-regulated autophagy modulator	DRAM	1.965	NM_018370	12q23.2
	Cyclin-dependent kinase inhibitor1A, transcriptvariant2	CDKN1A	2.937	NM_078467	6p21.31
	Lectin, galactoside-binding, soluble, 7 (galectin 7)	LGALS7	3.384	NM_002307	19q13.2
	Nuclear protein 1, transcript variant 2	NUPR1	2.818	NM_012385	16p11.2
	Insulin-like growth factor binding protein, transcript variant 1	IGFBP1	1.575	NM_000596	7p13
Epithelial mesenchymal	Keratin 19	KRT19	3.498	NM_002276	17q21.2
Transition	Caveolin 2, transcript variant 1	CAV2	1.57	NM_001233	7q31.2
	Cadherin 1, type 1, E-cadherin (epithelial)	CDH1	1.57	NM_004360	16q22.1
Tumor suppressors	Ras association (RalGDS/AF-6)domain family member10	RASSF10S100A4	1.522	NM_1080521	11p15.2
	S100 calcium binding protein A4, transcript variant 1	RB1	3.891	NM_002961	1q21.3
	Retinoblastoma 1		1.099	NM_000321	13q14.2
Oncogenes	v-ets erythroblastosis virus E26 oncogene homolog 1 (avian)	ETS1	1.583	NM_005238	11q24.3
	Jun oncogene	JUN	1.904	NM_002228	1p32.1

**Table 5:** Down -Regulated Genes in post HMGA2 silenced in Y79 cells (cDNA microarray analysis).

**Gene classification**	**Gene Description (Homo sapiens, gene, mRNA)**	**Gene symbol**	**Fold change**	**GeneBank accession**	**Chromosome location**
Angiogenesis	Fscin homolog 1, actin-bundling protein (S .rpuratus)	FSCN1	−1.43	NM_003088	17p13.3
Apoptosis	cDNA FLJ13706 fis, clone PLACE2000317	STAMBP	−2.611	NM_201647	18q23
	RRP5 protein homolog (Programmed cell death protein 11)	PDCD11	−1.24	NM_198490	3q21.3
Anti-apoptosis	G-2 and S-phase expressed 1	GTSE1	−1.089	NM_016426	Xp11.23
	High-mobility group box 1	HMGB1	−1.047	NM_002128	20q13.32
Cell cycle & differentiation	Cyclin-dependent kinase 6, mRNA	CDK6	−1.55	NM_001259	14q11.2
	CNDP dipeptidase 2 (metallopeptidase M20 family)	CNDP2	−1.084	NM_018235	9q33.3
Epithelial mesenchymal transition	Snail homolog 1 (Drosophila)	SNAI1	−1.155	NM_005985	11q13.1
	Transcription factor 4	TCF4	−1.313	NM_003199	8q24.21
Cell proliferation	Cyclin-dependent kinase 6	CDK6	−1.55	NM_001259	12q13.3
	Fibroblast growth factor receptor-like 1	FGFRL1	−1.822	NM_01004356	10q26.13
	c-src tyrosine kinase	CSK	−1.14	NM_004383	14q11.2
	Antigen identified by monoclonal antibody K_i_-67	MKI67	−2.378	NM_002417	17q21.32
	Cell division cycle 25 homolog B (S. pombe)	CDC25B	−1.319	NM_021873	1p34.1
	E2F transcription factor 4, p107/p130-binding	E2F4	−1.354	NM_001950	8p21.1
	Tumor protein p53 inducible protein 11	TP53I11	−1.143	NM_006034	2p13.1
Oncogene	v-abl Abelson murine leukemia viral oncogene homolog 1	ABL1	−1.167	NM_005157	22q13.2
	RAB22A, member RAS oncogene family	RAB22A	−1.554	NM_020673	6p21.33
	v-myc myelocytomatosis viral oncogene homolog (avian)	MYC	−1.526	NM_002467	12p13.33
	v-crk sarcoma virus CT10oncogene homolog (avian) transcript 2	CRK	−1.775	NM_016823	1p35.1
	v-myb myeloblastosis viral oncogene homolog (avian)-like 2	MYBL2	−1.231	NM_002466	21q22.11
	ELK1, member of ETS oncogene family	ELK1	−1.673	NM_005229	10q24.33
Tumor suppressors	Breakpoint cluster region, transcript variant 1	BCR	−1.428	NM_004327	22q13.31
	Tumor protein p53 inducible nuclear protein 2	TP53INP2	−1.301	NM_021202	11q14.1
Transcription factors	Multiple endocrine neoplasia, transcript variant e1E	MEN1	−1.526	NM_130803	12q13.13
	ELK1, member of ETS oncogene family	ELK1	−1.673	NM_005229	7q34

### Upregulation of genes modulated by the treatment of anti-*HMGA2* short interfering RNA in Y79 cells

In the Y79 cells, silencing of the *HMGA2* gene resulted in upregulating gene transcripts involved in the cellular functions, namely, the apoptosis genes—lactalbumin, alpha- (*LALBA*), phorbol-12-myristate-13-acetate-induced protein 1 (*PMAIP1*), insulin-like growth factor binding protein 1 (*IGFBP1*), IKK interacting protein (*IKIP*), tumor necrosis factor receptor superfamily, member 10b (*TNFRSF10B*), *Homo sapiens* receptor (*TNFRSF*)-interacting serine-threonine kinase 1 (*RIPK1*), *Homo sapiens* damage-regulated autophagy modulator (*DRAM*), ataxin 3 (*ATXN3*), mitogen-activated protein kinase 13 (*MAPK13*), *Homo sapiens* activating transcription factor 3 (ATF3), *Homo sapiens* nuclear protein 1 (*NUPR1*), *Homo sapiens* cyclin-dependent kinase inhibitor 1A (*p21, Cip1; CDKN1A*), lectin, galactoside-binding, soluble, 7 (*galectin 7; LGALS7*); cell cycle and differentiation genes—*Homo sapiens* 5-hydroxytryptamine (serotonin) receptor 5A (*HTR5A*), tachykinin 3 (*neuromedin K, neurokinin beta; TAC3*), *Homo sapiens* activating transcription factor 3 (*ATF3*); anti-proliferation—*Homo sapiens* maternally expressed 3 (*MEG3*); and cell adhesion—*Homo sapiens* cadherin 11, type 2, OB-cadherin (osteoblast; *CDH11*), *Homo sapiens* cadherin 1, type 1, E-cadherin (epithelial; *CDH1*), *Homo sapiens* integrin, alpha 1 (*ITGA1*), *Homo sapiens* integrin, alpha 2 (*ITGA2*), *Homo sapiens* laminin, alpha 3 (*LAMA3*), *Homo sapiens* laminin, beta 3 (*LAMB3*), *Homo sapiens* laminin, gamma 2 (*LAMC2*), *Homo sapiens* matrix metallopeptidase 2 (*MMP2*), *Homo sapiens* matrix metallopeptidase 9 (*MMP9*), and *Homo sapiens* collagen, type IV, alpha 3 (*COL4A3*).

### Downregulation of genes modulated by the treatment of anti-*HMGA2* short interfering RNA in Y79 cells

In the Y79 cells, silencing of the *HMGA2* gene resulted in downregulating gene transcripts such as the oncogenes— *Homo sapiens* v-abl Abelson murine leukemia viral oncogene homolog 1 (*ABL1*), *Homo sapiens* v-raf murine sarcoma 3611 viral oncogene homolog (*ARAF*), *Homo sapiens* v-crk sarcoma virus CT10 oncogene homolog (avian; *CRK*), *Homo sapiens ELK1*, member of ETS oncogene family (*ELK1*), *Homo sapiens* v-yes-1 Yamaguchi sarcoma viral related oncogene homolog (*LYN*), *Homo sapiens* v-myb myeloblastosis viral oncogene homolog (avian)-like 2 (*MYBL2*), *Homo sapiens* v-myc myelocytomatosis viral oncogene homolog (avian; *MYC*); cell cycle, proliferation, and differentiation genes—*Homo sapiens* transcription factor 4 (*TCF4*), *Homo sapiens* cyclin-dependent kinase 6 (*CDK6*), *Homo sapiens* cell division cycle 25 homolog A(*CDC25A*), *Homo sapiens* E2F transcription factor 4, p107/p130-binding (*E2F4*), *Homo sapiens* cyclin-dependent, *Homo sapiens* antigen identified by monoclonal antibody K_i_-67 (*MKI67*), *Homo sapiens* polo-like kinase 1 (*Drosophila*; *PLK1*)—*Homo sapiens* CNDP dipeptidase 2 (metallopeptidase M20 family; *CNDP2*), pre-B-cell leukemia transcription factor 1 (Homeobox protein *PBX1*), *Homo sapiens* snail homolog 1 (*Drosophila*; *SNAI1*); apoptosis genes—*Homo sapiens* cDNA FLJ13706 fis, clone, *Homo sapiens* T-box 3 (ulnar mammary syndrome; *TBX3*), transcript variant 2, *Homo sapiens* topoisomerase (DNA) II alpha; and angiogenesis genes—*Homo sapiens* fascin homolog 1, actin-bundling protein (*Strongylocentrotus purpuratus*; *FSCN1*), Antiapoptosis gene: *Homo sapiens* G-2 and S-phase expressed 1 (*GTSE1*).

### Deregulated pathways modulated by the treatment of the anti-*HMGA2* gene short interfering RNA in Y79 cells

**Figure 6:**
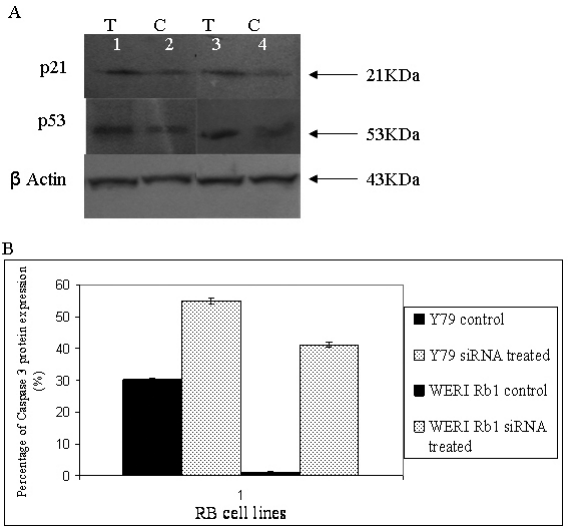
Effect of small interfering RNA on the expression of p21, p53, and caspase 3 in retinoblastoma (Y79, WERI Rb1) cells in vitro. **A**: western blot analysis demonstrates a marked increase in p21 and p53 expression in Y79 cells treated with *HMGA2*-siRNA (lane 1), and p21 and p53 expression of HMGA2 in non-transfected Y79 cells (lane 2), a marked increase in p21 and p53 expression in WERI Rb 1 cells treated with *HMGA2* siRNA (lane 3), and p21 and p53 expression in non-transfected WERI Rb1 cells (lane 4). **B**: The graphical representation of increased caspase 3 expression in *HMGA2* siRNA transfected RB cells is compared to the non-transfected RB cells with flow cytometric analyses.

In the current study, we found deregulation of genes associated with the mitogen-activated protein (MAP) kinase, Ras, Janus kinase/signal transducers and activators of transcription (Jak/STAT), and p53 signaling pathways. The downregulated genes involved in the MAP kinase pathway are *CRK, ELK1, MYC, CDC25B*, and *GRB2*. The downregulated genes involved in the Ras pathway are *Rac1, RALGDS*, and *ELK1*. The downregulated genes involved in the Jak/STAT pathway are *SPREAD2, PIAS3, CCND2*, and *CNTFR*. The increased levels of ATM, PUMA /BB3, PTEN, and DRAM, the downstream molecules of p53-mediated apoptosis, reveal the modulation of cell apoptosis. The role of p53-mediated apoptosis was confirmed by the overexpression of p53 and p21 proteins as well as the caspase 3 protein in *HMGA2*-silenced Y79 and WERI Rb1 cells (Figure 6A,B).

### Q-RT–PCR confirmation of microarray analysis in *HMGA2* silenced retinoblastoma cells (Y79 and WERI Rb1)

**Figure 7:**
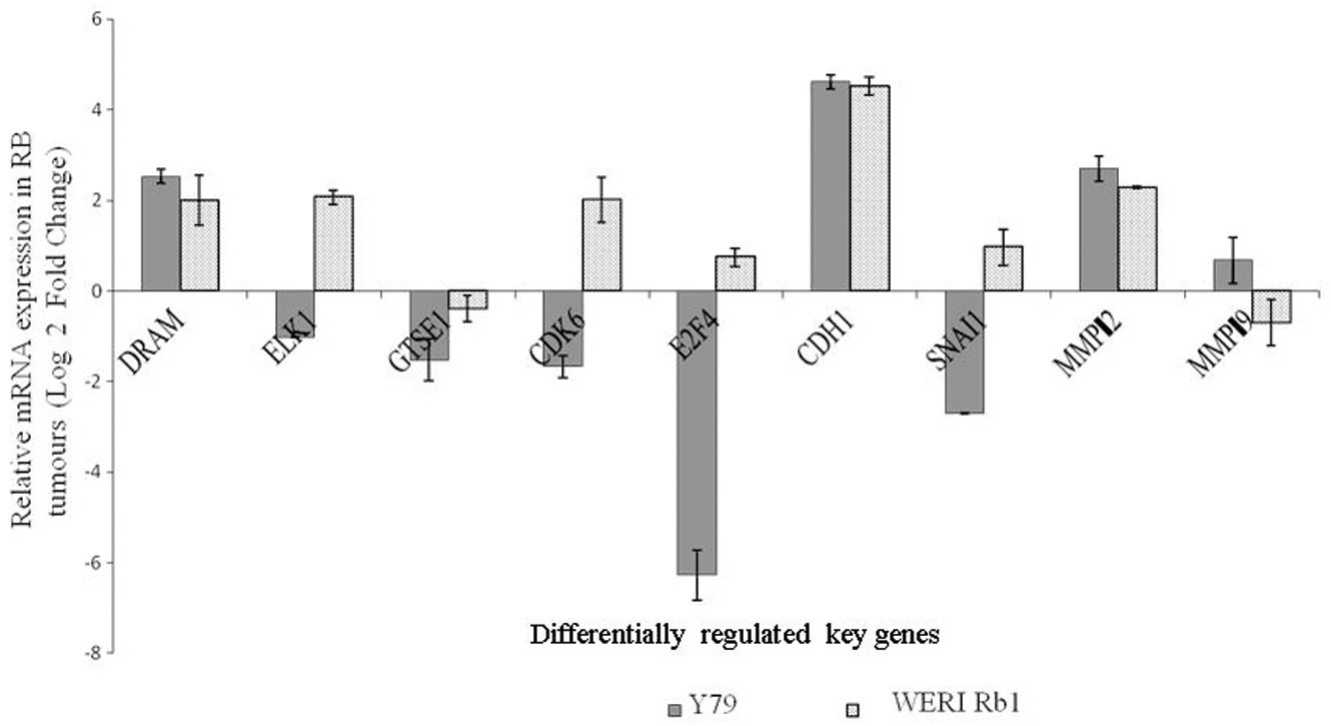
The mRNA expression of selected genes from the microarray data was confirmed using real-time quantitative reverse-transcriptase PCR. The black bars represent the mRNA levels quantified with qRT–PCR in the *HMGA2*-short interfering siRNA treated Y79 cells, and the spotted bars represent the fold expression of genes in the *HMGA2*-short interfering (siRNA) treated WERI Rb1 cells. The error bars represent the standard deviation of triplicate values. Abbreviations: DRAM represents damage-regulated autophagy modulator, ELK1: member of ETS oncogene family, GTSE1: G-2 and S-phase expressed 1, CDK6: cyclin-dependent kinase 6, E2F4:E2F transcription factor 4, p107/p130-binding, CDH1: cadherin 1, type 1, E-cadherin (epithelial; 1), SNAI1: snail homolog 1 (Drosophila), MMP2: matrix metallopeptidase 2, MMP 9: matrix metallopeptidase 9.

The gene expression level of nine genes (*ELK1, CDK6, E2F4, GTSE1, DRAM, CDH1, SNAI1, MMP2,* and *MMP9*) in the microarray analysis was consistent with the qRT–PCR findings in the transfected Y79 cells. Although most of the genes were consistent in the expression obtained with the microarray and qRT–PCR analyses, a few genes in the post-transfected WERI Rb1 cells differed in levels of expression with respect to microarray findings. These genes include *ELK1, CDK6,* and *E2F4*, which were not downregulated, unlike in the Y79 cells. The SNAI1 gene that was significantly downregulated in Y79 cells was not downregulated to the same extent in the *HMGA2*-silenced WERI Rb1 cells (that is, the expression level was not below the −1.0 cutoff value; Figure 7).

### Constitutive gene expression of deregulated genes in retinoblastoma primary tumors with qRT–PCR

**Figure 8:**
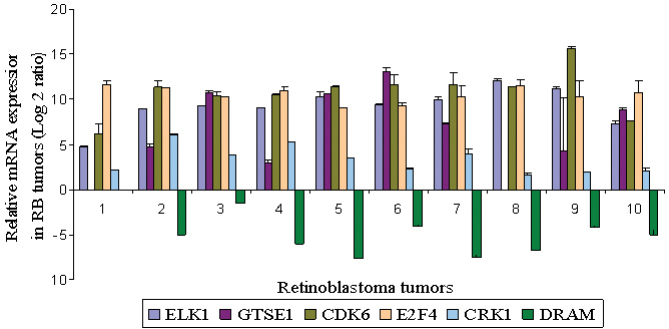
The constitutive mRNA expression of selected genes from microarray data in RB primary tumor tissues using real-time quantitative reverse-transcriptase PCR. The error bars represent the standard deviation of triplicate values. Abbreviations: ELK1: member of ETS oncogene family, GTSE1: G-2 and S-phase expressed 1, CDK6: cyclin-dependent kinase 6, E2F4:E2F transcription factor 4, p107/p130-binding, DRAM represents damage-regulated autophagy modulator, CDH1: cadherin 1, type 1, E-cadherin (epithelial; 1), SNAI1: snail homolog 1 (Drosophila).

The **e**xpression of the selected panel of genes (*ELK1, CDK6, E2F4, GTSE1, DRAM, CDH1,* and *SNAI1*) was compared for their relative expression in non-transfected primary RB tumors. We observed an inverse correlation of gene expression between the untransfected tumors and the *HMGA2*-silenced RB cells (Figure 8). For the ten RB tumor samples analyzed, the average levels of gene expression as follows: *ELK1* (9.21), *GTSE1* (6.23), *CDK6* (10.76), *E2F4* (10.51), *DRAM* (−4.79), *CDH1* (−0.430), and *SNAI1* (3.60), Table 2.

### Matrix metalloproteinase activity in the transfected Y79 and WERI Rb1 with zymography

**Figure 9:**
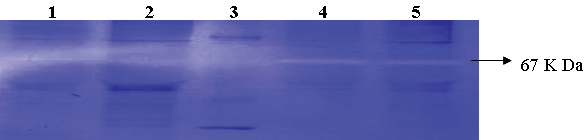
Effect of small interfering RNA on the expression of high mobility group A2 in retinoblastoma (Y79, WERI Rb1) cells in vitro. **A**: Zymography analysis demonstrates strong activation of MMPs in Y79 cells (lane 4), a mild increase in MMP activation in Y79 cells treated with *HMGA2*-siRNA (lane 5), strong activation of MMPs in WERI Rb1 cells (lane 2), and a mild decrease in MMP activation in WERI Rb1 cells treated with *HMGA2*-siRNA (lane 1), protein Molecular Weight ladder (lane 3).

Though there was increased expression of MMPs in post-transfected RB cells especially *MMP2* at the mRNA level, activity staining with zymography did not reveal a substantial difference between the pre- and post-transfected cells (Y79 showed a 5.6% increase and WERI Rb1 showed a 4.6% decrease compared to the control; Figure 9).

## Discussion

Chau et al. [9] reported that the HMGA2 protein contributed to the neoplastic transformation of retinal cells, and the authors mapped two transcription initiation sites and positive regulatory elements within the WERI-Rb1 cells. The findings of Chau et al. [9] suggested that *HMGA2* could become a therapeutic target, either by blocking HMGA2 protein expression in RB cells or by inhibiting expression of the *HMGA2* gene by targeting its promoters [9]. In the present study, we investigated the molecular pathways deregulated by HMGA2 in RB cells, by transient silencing of the *HMGA2* gene in in vitro models of RB (Y79 and WERI Rb1).

The cell cycle assay showed a marked transition in the G_1_/S phase with an increase in dead cell percentage. This also correlates with the significant upregulation of *p21/CDKN1A* (log transformed ratio=2.93), which is a direct target of miR-106b as it plays a key role in miR-106b-induced cell cycle growth [38].

HMGA2, as DNA binding proteins often referred to as architectural transcriptional factors, specifically interact with several transcription factors (NF-κB, ATF-2/c-Jun, Elf-1, Oct-2, Oct-6, SRF, NF-Y, PU-1, RAR) and participate in forming stereospecific multiprotein enhanceosome complexes [10]. Silencing the *HMGA2* gene in the RB cell lines (Y79, WERI Rb1) revealed deregulation of many functional genes. Their possible molecular mechanisms are discussed as follows.

The downregulation of *GTSE1* (G_2_ and S phase-expressed-1), a microtubule localized protein [39], in the *HMGA2* siRNA treated RB cells (Y79 and WERI Rb1) and its upregulation in primary RB tissues were validated. The GTSE-1 protein negatively regulates *p53* transactivation and p53-dependent apoptosis. The inhibition of *HMGA2* gene expression in RB cells inhibited cell proliferation, which corroborates with earlier studies in the nude mice model of RB [9]. This study reveals the upregulation of the apoptotic genes, namely, *DRAM*, damage-regulated autophagy modulator, a critical effector of p53- induced autophagy [40] in post-*HMGA2* silenced RB cells (Y79 and WERI Rb1). The constitutive expression of DRAM was downregulated in primary RB tumors. The upregulation of other p53-regulated genes involved in initiating apoptosis such as *ATM, PUMA/BB3, NOXA, FOXO4,* and *PTEN* (from the whole genome cDNA microarray analysis of HMGA2 siRNA treated Y79 cells) is indicative of p53-dependent apoptosis [41]. This was further validated by the observed protein overexpression of p53, p21, and caspase 3 in post-transfected Y79 and WERI Rb1 cells (Figure 6A,B). The induction of PUMA by the downstream molecules of TNF-α mediated apoptosis may mediate p53-independent apoptosis. The genes involved in this pathway are *ATF* and *CREB* families, which are increased in the post-*HMGA2* silenced RB cells [42]. Taken together, p53-dependent and -independent pathways seem to be involved in inducing apoptosis of *HMGA2*-silenced RB cells.

The study also shows the downregulation of various transcription factors and cyclin-dependent kinases involved in cell cycle regulation, namely, *E2F4* and *CDK6* in *HMGA2* post-silenced Y79 cells. Cyclin-dependent kinases (CDKs) are important regulators of cell cycle progression. CDK6, which first appears in the mid-G_1_ phase, is important for G_1_ phase progression and G_1_/S transition. Coupled with CDK4, they negatively regulate the activity of the RB tumor suppressor protein (The PubMatrix database). Exit from the G_1_ phase of cell cycle division is regulated by phosphorylation of pRb by cyclin D/CDK4 and cyclin D/CDK6 complexes. This results in the suppression of the cell cycle. The constitutive expression of *E2F4* and *CDK6* genes was increased in our study cohort of RB tumor tissues analyzed here (Figure 7). Another E2F family transcription factor, E2F3, was earlier reported to be overexpressed in RB by Gallie et al. [43]. Increased expression of CDK6 in other primary tumors such as in squamous cell carcinoma [44,45], basal cell carcinoma [46], medulloblastoma, and B-cell lymphoproliferative disorder [47] have been reported in which increased CDK6 expression has been correlated with induced cell proliferation and malignant transformation coupled with cyclins. Thus, the significant downregulation of CDK6 coupled with the cyclins, CCND2 and E2F4, in post-*HMGA2* silenced Y79 cells substantiates the suppression of cell proliferation. There are a few differences in the gene expression of *E2F4, CDK6,* and *ELK1* between the two RB cell lines (Y79, WERI Rb1). This is possibly because of the differential activation and dominance of specific apoptotic and cell cycle pathways that may relate to tumor aggressiveness. WERI Rb1 represents a non-metastatic model of RB while Y79 represents more aggressive and metastatic characteristic of RB [48].

The other gene modulations in the current study include the significant downregulation of *SNAI1* (Snail, a transcriptional repressor of E-cadherin) along with simultaneous upregulation of E-cadherin in the post-anti-*HMGA2* silenced RB cells. *HMGA2* silencing induced transcriptional derepression of E-cadherin with decreased Snail has been reported in a pancreatic cancer cell line by Sugiko et al. [49]. The constitutive expression of *SNAI1* and *CDH1* in RB tumor tissues revealed the inverse correlation between them (Figure 7). This has been confirmed in hepatocellular carcinoma [50], oral squamous cell carcinoma [51], melanoma [52], and breast carcinoma [53]. Increased Snail expression resulted in tumor progression and metastasis in MDA-MB-231 cells [54], mouse skin carcinoma cell lines [55], and tongue squamous cell carcinoma [56]. This deregulation of Snail and E-cadherin may contribute to the upregulation of the extracellular matrixes such as laminins α3, β3, γ2 (laminin 5: LN 5), and type IV collagen, and downregulation of laminin α5 and integrin α5. The enhanced expression of laminins, especially laminin 5 and type IV collagen observed here in the *HMGA2* silenced Y79 cells, may indicate decreased cell detachment [57]. The present finding of SNAI’s regulation in the expression of laminins, integrins, and other extracellular matrix proteins supports the role of SNAI’s cell adhesion mechanisms involved in cancer progression. Thus, Snail-mediated modulation of ECM proteins serves as one of the mechanisms by which cancer progression is controlled by *HMGA2* silencing [58,59]. Matrix metalloproteinases (MMPs) have a role in tumor progression that is determined by a balance of its activators and inhibitors [60–64]. In the present study, the increase in the expression of some MMPs in the treated cancer cells may not be sustained, as their positive and negative regulators do not show any significant increase or decrease in their expression. Upregulation of MMPs (*MMP2, MMP3, MMP7,* and *MMP9*) with no significant change in the expression of their inhibitors (TIMPs) was observed in post-*HMGA2* silenced RB cells. The microarray analysis did not reveal significant changes in the levels of thrombospondin-2 (activator of MMP2) and thrombospondin-1 (activator of MMP9) [51,52]. Thus, the increased level of MMPs is not associated with the corresponding increase in their activators. This is further confirmed by our zymographic assay of MMP activity that revealed only a mild change in MMP activity in the HMGA2-silenced RB cells compared with control cells. In the post-HMGA2 silenced Y79 cells, a 5.6% increase in MMP activity while a 4.6% decrease in MMP activity in WERI Rb1 cells was observed relative to control cells.

In conclusion, *HMGA2* silencing in RB cancer cells resulted in the deregulation of genes responsible for apoptotic, cell cycle, and cell adhesion mechanisms, thereby explaining the mechanisms by which cancer cell progression is suppressed in *HMGA2*-silenced RB cells. These findings are further substantiated by the inverse correlations between the deregulated gene expression in the *HMGA2*-silenced RB cells and in the primary RB tumor tissues. The *HMGA2* gene silencing approach is thus suggested to be a promising strategy in RB therapy.
